# Quantitative assessment of gene expression network module-validation methods

**DOI:** 10.1038/srep15258

**Published:** 2015-10-16

**Authors:** Bing Li, Yingying Zhang, Yanan Yu, Pengqian Wang, Yongcheng Wang, Zhong Wang, Yongyan Wang

**Affiliations:** 1 Institute of Basic Research in Clinical Medicine, China Academy of Chinese Medical Sciences, 16 Nanxiaojie, Dongzhimennei, Beijing 100700, China; 2 Institute of Information on Traditional Chinese Medicine, China Academy of Chinese Medical Sciences, 16 Nanxiaojie, Dongzhimennei, Beijing 100700, China; 3 Brightech International, LLC, 285 Davidson Ave #504, Somerset, NJ 08873, USA

## Abstract

Validation of pluripotent modules in diverse networks holds enormous potential for systems biology and network pharmacology. An arising challenge is how to assess the accuracy of discovering all potential modules from multi-omic networks and validating their architectural characteristics based on innovative computational methods beyond function enrichment and biological validation. To display the framework progress in this domain, we systematically divided the existing *Computational Validation Approaches based on Modular Architecture (CVAMA)* into topology-based approaches (TBA) and statistics-based approaches (SBA). We compared the available module validation methods based on 11 gene expression datasets, and partially consistent results in the form of homogeneous models were obtained with each individual approach, whereas discrepant contradictory results were found between TBA and SBA. The TBA of the Zsummary value had a higher Validation Success Ratio (VSR) (51%) and a higher Fluctuation Ratio (FR) (80.92%), whereas the SBA of the approximately unbiased (AU) p-value had a lower VSR (12.3%) and a lower FR (45.84%). The Gray area simulated study revealed a consistent result for these two models and indicated a lower Variation Ratio (VR) (8.10%) of TBA at 6 simulated levels. Despite facing many novel challenges and evidence limitations, *CVAMA* may offer novel insights into modular networks.

Modularity is a common characteristic of omics-based biological networks^1^,^2^,^3^. Module-based analyses that investigate or deconstruct omics-based biological networks have become a hot topic in recent years^4^,^5^. Various types of algorithms have been proposed to identify modules (also known as communities, clusters, and subnetworks), including network clustering^6^,^7^, heuristic search^8^,^9^, seed extension^10^, topology network^11^,^12^, and matrix decomposition^13^,^14^. However, in contrast to the large number of module detection methods^4^, there are few methods for module validation and evaluation. How to evaluate the accuracy and validity of modules has become a new challenge for researchers. Most previous studies used function enrichment methods to evaluate modules based on functional annotations, such as GO, MIPS, and KEGG^15^,^16^,^17^,^18^,^19^,^20^,^21^,^22^,^23^,^24^. However, some modules may be enriched with too many functions, whereas others may be enriched without any functions, and the background annotation database itself is constantly being updated. Other studies used molecular biological experimental techniques to verify the co-expression, transcription regulation or other interaction relationships among members of a given module^25^,^26^,^27^,^28^,^29^,^30^. However, this method is only suitable for small modules that consist of only a few nodes, and it is nearly impossible to perform this method for a larger module. Thus, in the era of Big Data and omics revolution, an arising challenge is to explore rational strategies to validate biological network modules.

Several published studies have employed *Computational Validation Approaches based on Modular Architecture* (*CVAMA*) to evaluate modules’ authenticity, reproducibility, and significance or to identify phenotype-related functional modules^31^,^32^,^33^,^34^,^35^. These approaches are not limited by module size and supporting databases. With an increasing number of omics technologies and module analysis methods, *CVAMA* may become the new focus. In this paper, we summarized the available *CVAMA* methods, which were divided into topology-based approaches (TBA) and statistics-based approaches (SBA). One representative method of each was selected to validate modules obtained from genomic datasets, and comparative analyses were performed to illuminate the feasibility and challenges in *CVAMA*.

## Results

### Topology-based approaches (TBA) for module validation

A module may have several topological features, such as modularity^2^, connectivity^36^, density^36^,^37^, clustering coefficient^37^, degree^38^, and edge betweenness^39^. Module detection methods may focus on one or a few topological criteria, and it is also essential to determine whether the identified modules have a modular structure. Therefore, we may use a single or composite topological index to evaluate whether a module is valid (Table 1). Any single topological index used to validate a module should be independent of the methods used to identify the module, such as the network perplexity index of *Entropy*^40^,^41^. The entropy increases when the data are more uniformly distributed; therefore, a good quality module is expected to have a low entropy^42^,^43^. Topological indexes, including intra-modular connectivity^44^ and *NB* value^26^, have been applied to evaluate whether the intra-modular structure is different from other parts of the whole network. Other indexes, such as compactness^45^ and weak community^46^, can be used to select good clusters from integrated clustering results. Because a single topological index is not likely to provide a global evaluation of the modular structure, an alternative choice is to combine multiple topological indexes into an integrated measure to assess a module’s validity. Both internal and external indexes, such as density, connectivity, and tabulation-based module preservation statistics, can be integrated to validate the existence of a module^35^,^47^. Based on a global view of the modular structure, it may be advantageous to aggregate multiple module evaluation statistics into summary preservation statistics. In our study, we validated five preserved modules whose *Zsummary* value (an integrated index) was greater than 2.

### Statistics-based approaches (SBA) for module validation

In addition to topological criteria, a module should also be statistically significant, which means that the modular architecture distribution ought to be highly unlikely to be obtained by chance in a randomized network. Moreover, exploring the relationship between modules and various phenotypes or identifying consistent modules may also require significance testing^31^,^33^,^48^,^49^. For this reason, SBA is an important process to assess a module’s stability, phenotypic correlation or significance of consistency (Table 1). For responsive modules or module biomarker identification^50^,^51^,^52^, binary or mixed integer linear programming models can be used to validate the causal or dependent relations between network modules and biological phenotypes^34^,^53^,^54^. In phylogeny, resampling approaches are defined as a confidence measure for splits in a phylogenetic tree and are used to calculate consensus trees^55^, which can also be used to assess the robustness of modules in network analysis^25^,^56^. A permutation test with a p value calculated by empirically estimating the null distribution can be adopted to determine whether the module composition is higher than expected by chance or associated with the disease being investigated^57^,^58^. Moreover, given two or more networks, comparative network analysis is often used to identify modules across networks or species, and these modules are defined as consensus or conserved modules^31^,^59^. Moreover, a module’s “reproducibility” can also be assessed, i.e., to what extent a module obtained from one network is compatible with modules in another network^46^,^55^,^60^,^61^,^62^. In our study, by using hierarchical clustering, we identified 66 statistically significant modules based on approximately unbiased (AU) p-values.

Module identification based simply on topological criteria or statistical significance may not discover certain types of biologically meaningful modules^63^. Because disparate results can be obtained from the same network with different algorithms, functional validation can be used to evaluate the performance of different module identification methods^64^. Although it is not our focus in this study, we summarize and list functional module validation methods reported in the published literature (Supplementary Table 1). Typically, the most widely used functional validation method is functional homogeneity evaluation^65^,^66^, with indexes such as functional enriched p value^7^,^45^,^67^ and R score^7^,^68^. Furthermore, the index of quantitative score based on function enrichment analysis may be applied to assess a module’s confidence level^21^ or disease relationship^69^. Moreover, known protein complex matching can also provide functional evidence for modules, and the commonly used indexes include the overlapping score (OS)^7^,^70^ and F-measure^67^,^71^. Other measurements, such as the positive predictive value (PPV), accuracy, and separation, can also be used^72^. For small modules, the experimental techniques of molecular biology, such as real-time quantitative PCR (qPCR), western blotting, and siRNA knock-down, may be applied to validate the co-expression, co-regulation or other interaction relations among the genes or proteins within a module^25^,^26^,^27^,^28^.

### Homogeneity of different models of TBA on the same dataset

**Figure 1:**
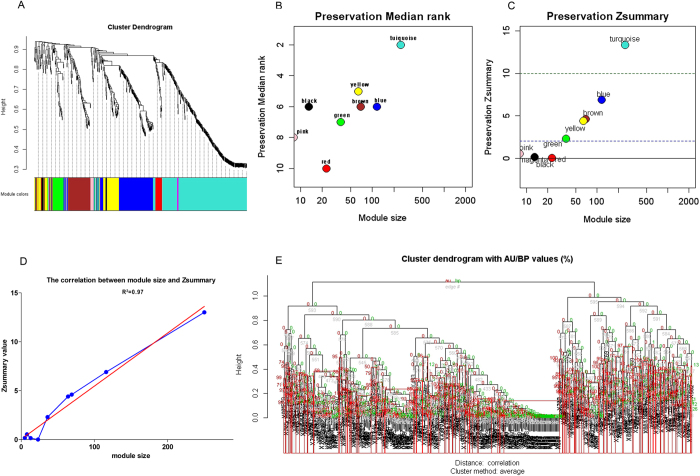
(**A**) Hierarchical cluster tree showing coexpression modules identified by WGCNA. Each leaf in the tree represents one gene. The major tree branches constitute 9 modules labeled by different colors. (**B**) The medianRank preservation statistics (y-axis) of the modules. Each point represents a module, labeled by color and names. Low numbers on the y-axis indicate high preservation. (**C**) The Zsummary preservation statistics (y-axis) of the modules. The modules are labeled as in panel (**B**) The dashed blue and green lines indicate the thresholds. A Zsummary value over 2 represents a moderately preserved module, and a value over 10 provides strong evidence of module preservation. (**D**) Scatter plots showing the correlation between the Zsummary (y-axis) and module size (x-axis). (**E**) The cluster dendrogram with approximately unbiased (AU) P-values. The AU p-values are displayed in red, and clusters with an AU p-value lower than 0.05 are highlighted by rectangles. The calculations and the drawn figure were performed using the pvclust R package.

Both *Zsummary* and *medianRank* are integrated topological indexes of module preservation. We applied these two models to evaluate modules identified from the same dataset (GSE24001), which was derived from 30 newly diagnosed infant acute lymphoblastic leukemia samples. Modules were identified by the Weighted Gene Co-Expression Network Analysis (WGCNA) R package^73^, setting 3 as the minimum module gene number. Each module was detected based on a hierarchical cluster tree and was labeled by colors (Fig. 1A). The validation results are shown in Fig. 1B,C. Five preserved modules (*Zsummary* ≥ 2) all had a relatively low *medianRank* values, and the most strongly preserved module (*Zsummary* = 13) had the lowest *medianRank* value. Similarly, the two modules that had the highest *medianRank* value (*medianRank* = 10) were both unpreserved.

### Example modules validated by two models of TBA

**Figure 2:**
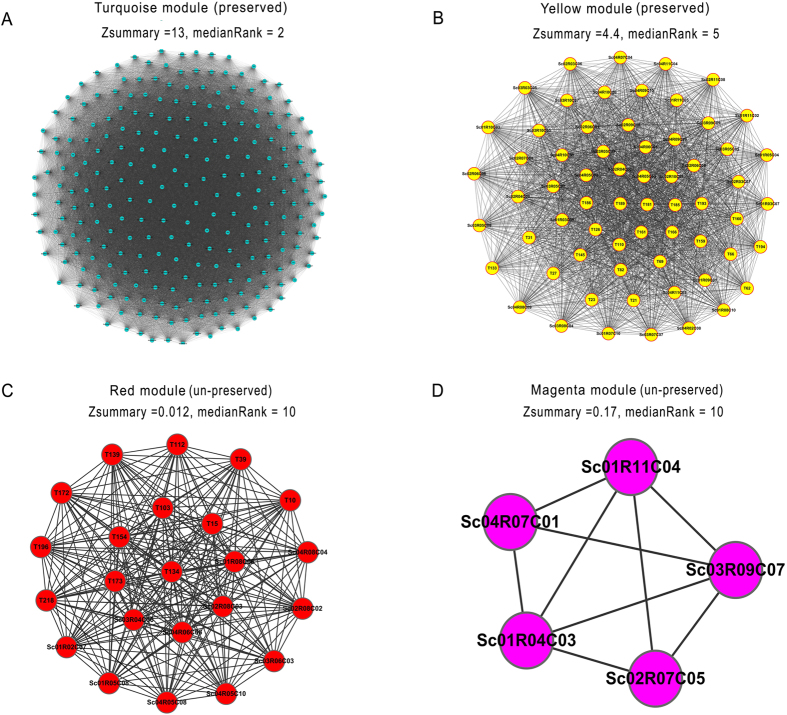
The preserved and unpreserved modules. Each node is a gene, and each edge is the co-expression relationship. (**A**) The turquoise module. (**B**) The yellow module. (**C**) The red module. (**D**) The magenta module.

Among the 5 preserved modules, the turquoise module, which had 250 nodes and 30,833 edges, was most strongly preserved (*Zsummary* = 13), having the lowest *medianRank* value (*medianRank* = 2) (Fig. 2A). The preserved yellow module, which was composed of 64 nodes and 1,970 edges, had a low *medianRank* value (*medianRank* = 5) (Fig. 2B). Among the 4 unpreserved modules, the red module, consisting of 23 nodes and 253 edges (Fig. 2C), and the magenta module, consisting of 5 nodes and 9 edges (Fig. 2D), had the highest *medianRank* values (both *medianRank* = 10).

### Variability of TBA (*Zsummary*) and SBA (AU *P-value*) results on the same dataset

Based on the same dataset (GSE24001), we compared the validation results of TBA and SBA, choosing *Zsummary* and *AU p-value* as the representative methods for each approach. For *Zsummary*, 5 preserved modules were validated from 9 modules. The *Zsummary* value was dependent on the module size, which was consistent with previous studies^35^ (Fig. 1D). The *AU p-value* used to access the modular architecture distributional probability was computed to search for significant modules (clusters). As shown in Fig. 1E, 66 modules with 3 or more genes and an *AU p-value* larger than 0.95 are highlighted by rectangles, which are strongly supported by the gene expression data. Different numbers of detected modules and valid modules were obtained through the two methods.

### Multiple comparisons of *Zsummary* and *medianRank* on 10 datasets

**Figure 3:**
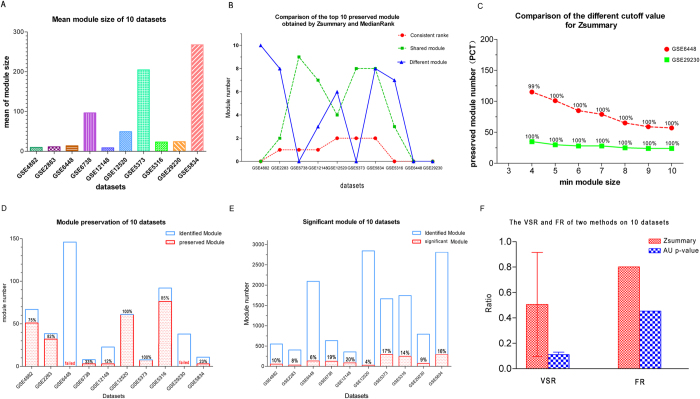
The comparison of TBA (Zsummary and medianRank) and SBA (AU p-value) on 10 datasets. (**A**) A mean module size of 10 datasets. The y-axis is the mean module size (nodes), and the x-axis is each dataset number. (**B**) Comparison of the top 10 preserved modules validated by Zsummary and medianRank. The red spots represent the number of consistently ranked modules. The green spots represent the number of overlapping modules. The blue spots represent the number of non-overlapping modules. (**C**) The effect of changing the minimum module size setting from 4 to 10 on two datasets. The y-axis is the percentage of preserved modules, and the x-axis is the different cutoff value settings. (**D**) The percentage of preserved modules validated by Zsummary on 10 datasets. (**E**) The percentage of significant modules validated by AU P-value on 10 datasets. In (**D,E**) the blue bars indicate the number of all the modules detected, and the red bars indicate the number of preserved or significant modules. (**F**) The VSR and FR of Zsummary and AU P-value on 10 datasets. The red bars represent Zsummary, and the blue bars represent the AU P-value.

For the same modules identified by WCGNA, we applied the integrated topological indexes *Zsummary* and *medianRank* to 10 datasets to further compare the results of module preservation evaluation. The average size of modules obtained from the 10 datasets is shown in Fig. 3A. Because *medianRank* was a relative preservation index without a cutoff value, we compared the top 10 ranked modules validated by *Zsummary* and *medianRank* (Fig. 3B). We failed to obtain a valid *Zsummary* value in two datasets (GSE6448, GSE29230) when the minimum module size was set at 3. For the other 8 datasets, overlapping preserved modules validated by *Zsummary* and *medianRank* were found in 7 datasets, and consistent ranked modules were observed in 6 datasets, demonstrating the consistency of the two indexes. However, no overlapping modules in the top 10 preserved modules were found in one dataset (GSE4882).

### *Zsummary* analysis was impeded by small module size

Because we failed to obtain a valid Zsummary value in two datasets (GSE6448, GSE29230) with a minimum module size of 3, we changed this cutoff value from 4 to 10. Then, valid Zsummary values were acquired in both datasets, and the percent of preserved modules was stable (Fig. 3C) due to the too small density or connectivity, leading to invalid *Zsummary* values when the minimum module size was set at 3.

### Comparison of TBA (*Zsummary*) and SBA (AU *P-value*) results for 10 datasets

As mentioned above, *Zsummary* is a TBA index and *AU P-value* is an SBA index. Based on 10 datasets, we compared the performance of these two types of index. In this application, modules with 3 or more genes were considered as valid modules. The proportions of valid (preserved or significant) modules obtained by these two indexes from 10 datasets are shown in Fig. 3D,E. For different methods and datasets, both the module number and the proportion of valid modules varied greatly. Overall, the Validation Success Ratios (**VSR**) of *Zsummary* and *AU P-value* were 51% and 12.3%, respectively (Fig. 3F). This indicated that *Zsummary* obtained a higher ratio of valid modules (invalid values from two datasets were deemed as zero). A prior study adopted *Zsummary* to validate CASTxB6 female liver modules with 9 other expression datasets and revealed an average **VSR** of 86.44%^74^. However, *Zsummary* also had a higher Fluctuation Ratio (**FR**, 80.92%) than *AU P-value* (45.84%), indicating that the stability of the *AU P-value* results was superior to that of Zsummary (Fig. 3F).

### Correlation between the network parameters and the ratio of valid modules

**Figure 4:**
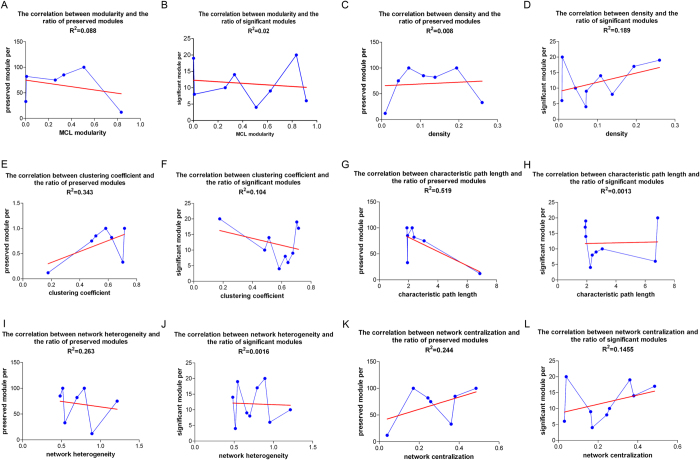
The relationship between the percentage of valid (preserved or significant) modules (y-axis) and the network parameters (x-axis). The network parameters were calculated by plugins in Cytoscape. (**A,B**) MCL versus valid module percentage. (**C,D**) Density versus valid module percentage. (**E,F**) Clustering coefficient versus valid module percentage. (**G,H**) Characteristic path length versus valid module percentage. (**I,J**) Network heterogeneity versus valid module percentage. (**K,L**) Network centralization versus valid module percentage. The red line added to each plot is the linear regression line with intercept 0 and slope 1.

To further determine which network parameters influence the ratio of valid modules of the *Zsummary* and *AU P-value* methods, we selected 6 main network parameters of the 10 datasets (Supplementary Table 2), i.e., modularity, density, clustering coefficient, characteristic path length, network heterogeneity, and network centralization. Linear regression analysis indicated that none of these 6 parameters was correlated with the valid module ratio of the *Zsummary* and *AU P-value* methods (Fig. 4). This implied that the valid module ratio of genomic networks may not be influenced by a single network parameter.

### Impact of gray area variation on 8 datasets

**Figure 5:**
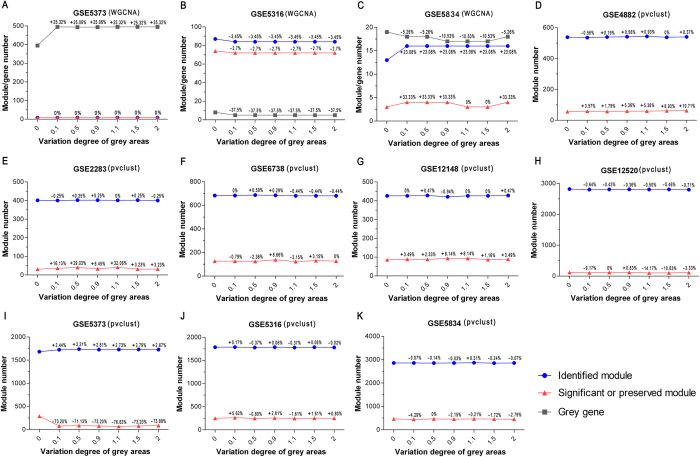
The changes of modules or gray genes (only for WGCNA) on 8 datasets by Gray area simulation. The blue spots represent the number of modules identified by WGCNA or pvclust. The red spots represent the number of valid (preserved or significant) modules. The gray spots are the number of gray genes. The numerical value above the spots is the VR at 6 simulated levels compared with the original dataset.

The gray area was the region of gray genes that was not assigned into any module and labeled in gray by WGCNA. Except for the two datasets (GSE6448 and GSE29230) without valid *Zsummary* values, 8 datasets were simulated by changing the gray area genes’ expression levels to 0.1, 0.5, 0.9, 1.1, 1.5, and 2 times that of the original dataset. Based on each simulated dataset, WGCNA (an R package used to compute *Zsummary*) and pvclust (an R package used to compute *AU p-value*) were performed for module identification and validation. For WGCNA, 5 datasets (GSE2283, GSE12148, GSE6738, GSE12520, and GSE4882) had no changes at the 6 simulated levels compared with the original datasets (a change in *Zsummary* less than the cutoff value was considered as no change). The changes in the number of modules or gray genes (only for WGCNA) and Variation Ratio (VR) for the remaining 3 datasets are shown in Fig. 5A–C. For pvclust, changes were observed in all 8 datasets compared with the original datasets, and the changes in the module number and VR are shown in Fig. 5D–K. No correlation was found between the VR and the simulated levels in the changed datasets.

### Comparison of TBA and SBA by Gray area simulation

**Figure 6:**
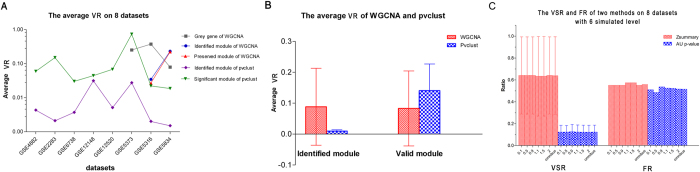
Comparison of WGCNA (Zsummary) and pvclust (AU p-value) on Grey area simulated datasets. (**A**) The average VR of the modules and genes of 8 datasets. (**B**) The average VR of WGCNA and pvclust of all simulated datasets. The red bars represent WGCNA, and the blue bars represent pvclust. (**C**) The VSR and FR of Zsummary and AU P-value of 8 datasets at 6 simulated levels. The red bars represent Zsummary, and the blue bars represent AU P-value.

For the 8 datasets, the average VRs of WGCNA and pvclust at the 6 simulated levels are shown in Fig. 6A. With regard to WGCNA, only the VRs in the changed datasets were calculated. The VRs of WGCNA and pvclust in all simulated datasets can be seen in Fig. 6B. WGCNA had a higher VR (8.43%) for module identification but a lower VR (8.10%) for module validation. By contrast, pvclust had a lower VR (1.29%) for module identification and a higher VR (14.06%) for module validation. Thus, the gray area changes had different impacts on the two models in terms of module identification and validation. Moreover, the VSR and FR of TBA (*Zsummary*) and SBA (*AU p-value*) were stable at each simulated level (Fig. 6C). *Zsummary* had a higher VSR and FR at each simulated level (2 datasets with invalid *Zsummary* values were not included). When data at all 6 simulated levels were aggregated, the VSR of *Zsummary* and *AU p-value* was 63.82% and 12.30%, and the FR was 55.84% and 51.42%, respectively.

## Discussion

Functional enrichment and biological experiments based on module validation methods may not satisfy the rising demands of various omics networks. As an alternative choice, *CVAMA* can potentially provide an analytical assessment of the structure and stability of modules captured by various partitioning methods and should be considered as a crucial tool in the interpretation of network modules. The feasibility of *CVAMA* was demonstrated in our applications of TBA and SBA-based module validation methods in genomic network modules. As a TBA, *Zsummary* had a high VSR (51%) but also a high FR (80.92%) and was impeded by small module size. As an SBA, *AU p-value* had a low FR (45.84%) but also a low VSR (12.3%). The Gray area simulated study showed that the VSR and FR of both TBA (Zsummary) and SBA (AU p-value) remained stable at each simulated level. Meanwhile, TBA (*Zsummary*) had a lower VR (8.10%) for module validation at the 6 simulated levels, indicating that the gray gene changes had little impact on the topology-based models. Although different validation results were obtained by the two types of method with different gene expression datasets and module detection methods, one may choose an appropriate validation index based on the topological structure or stability of the modules. For example, if we focus on whether a module is structurally different from the rest of the network, we may use *Zsummary* to assess its preservation. If we focus on whether a module is stable or robust, we may choose *AU P-value* to assess its confidence. Taken together, the existing methods are not ideal, and further improvement is justified.

Modular analysis in genomic networks is a complicated process that involves various factors. In general, there is no “golden standard” for assessing the validity and quality of modules, and different algorithms for module identification with different parameters may produce disparate module partition results^4^,^75^,^76^. Thus, it is difficult to determine which module is “correct” and which module partition method outperforms others. As our application demonstrated, different types of module validation indexes for the same network may generate different outcomes. Generally speaking, each type of module validation method may have its own advantages and disadvantages, and some methods may require certain conditions (e.g., data type, network pattern, or module identification method), limiting their applicability and flexibility. There is no universally acceptable approach that can perform well for all types of data under all scenarios, which results in challenges to make a clear-cut prescription for genomic module validation.

As for the possible discordance between topological criteria and biological meaning in module identification^77^, methods combining both function and structure have been proposed to identify functional modules^78^. Similarly, function and topology are also the two aspects of module validation. *CVAMA* may neglect the biological meaning and directly assess the correlation of modules with known functional annotation, which may deviate from the densely connected property. It is assumed that fusion of functional and topological evaluation may lead to a high quality selection of better modules. However, most of the existing methods in published literature focus on either function or topology, and researchers may only be interested in their own subject or module identification algorithm. Therefore, module validation methods that integrate both functional and topological indexes and are independent of the vagaries of module detection algorithms need to be further explored.

For the modules obtained by clustering algorithms, internal and external cluster validation methods for assessing the quality of clustering results have been discussed in previous studies^47^,^79^. The internal validation attempts to measure how well a given partitioning corresponds to the natural cluster structure of the data, and such indexes include compactness, connectedness, separation, combinations, and stability^41^. External validation attempts to compare the recovered structure to a priori knowledge and to quantify the match between them, and such indexes include unary measures and binary measures^79^. In addition to cluster quality assessment, how to estimate the optimal number of clusters has also been discussed^40^,^80^. Generally, if a module is known to be consistent with the known knowledge, it would show stronger evidence of preservation than a module without a priori evidence, such as a known pathway or co-transcriptional regulation^17^,^81^,^82^,^83^.

Gene interactions are dynamic in regulating the functioning of cells and organisms^84^,^85^. A number of studies have focused on dynamic module identification, as well as the dynamic behavior of modules in networks^11^,^86^,^87^. As such, module validation should not be constrained to a static situation. Modular dynamics may involve time-series molecular interaction^88^, environment changes^89^, phenotypic changes^90^,^91^, and ontogenetic and phylogenetic time^92^,^93^. Dynamic evaluation of modules in certain dynamic processes may generate more comprehensive results, which cannot be obtained in a static state. The more meaningful consequence is intertwined with greater challenges in dynamic *CVAMA*.

Therefore, to address the challenge of omics-based module validation, computing-based methods are an easy and feasible choice. Despite these challenges, *CVAMA*, in addition to functional enrichment and biological experiments, offers novel insights into module network research and may become a new paradigm in modular analysis.

## Materials and Methods

### Datasets and samples

Gene expression datasets were obtained from the GEO database (http://www.ncbi.nlm.nih.gov/geo/). Eleven spotted DNA/cDNA datasets from different organisms and experiment platforms were downloaded, with the sample size ranging from 28 to 592, and the gene number ranging from 448 to 3,520. Because a test dataset was needed for *Zsummary* and *medianRank,* we selected half of the samples in each dataset as reference data and the other half as test data. The raw gene-expression information is shown in Supplementary Table 2.

### Network construction and network parameter calculation

For each genomic dataset, weighted gene co-expression network analysis (WGCNA)^73^ was used to construct a network and to detect modules. The freely available WGCNA R package and R tutorials were described in^73^. After network construction, we exported each network to Cytoscape software^94^. Network analysis was conducted with the NetworkAnalyzer^95^ plugin to calculate the network topological parameters. Network modularity was calculated by the CommFinder^96^ plugin in Cytoscape. The network topological parameters and modularity are listed in Supplementary Table 2.

### *Zsummary* and *medianRank* module preservation statistics

*Zsummary* is an integrated statistic implemented in functional module preservation in the WGCNA R package^35^. It is composed of 4 statistics related to density and 3 statistics related to connectivity that can quantitatively assess whether the density and connectivity patterns of modules defined in a reference dataset are preserved in a test dataset. A *Zsummary* value between 2 and 10 indicates moderate module preservation, whereas a *Zsummary* > 10 provides strong support for module preservation^35^. Another integrated index, *medianRank*^35^, is also composed of statistics related to density and connectivity. It is a rank-based measure to compare the relative preservation among multiple modules; a module with lower *medianRank* tends to exhibit stronger observed preservation.

### Approximately unbiased (AU) p-value

The *AU p-value*, computed by multi-scale bootstrap resampling^97^, was selected as a representative SBA index. The *AU p-value* is often used to assess the uncertainty of clustering analysis^98^. In our application, the *AU p-value* was computed using the R package pvclust^99^ with 1,000 times resampling, varying the bootstrap sample size from 0.5 to 1.4-fold the real sample size of the gene expression data. We set clusters with an *AU p-value* larger than 0.95 as significant modules^99^.

### Validation success ratio (VSR) and fluctuation ratio (FR)

The VSR and FR were defined for comparing the two types of module validation approaches on multiple datasets. The VSR (Eq. 1) was defined as the average percentage of valid modules against all modules available on multiple datasets. The FR (Eq. 2) was defined as the stability or variation degree of the percentage of valid modules on multiple datasets; a lower FR indicated that the percentage of valid modules was more stable.


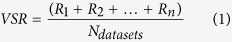



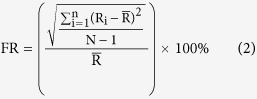


where *R* is the ratio of valid modules on one dataset, and *N* is the number of all available datasets.

### Simulated comparisons by changing the gray area gene expression level

In WGCNA, genes that were not assigned into any module were labeled in gray. We called these gray gene regions the gray area. The gray area represented genes whose profiles were simulated to be independent (i.e., without any correlation structure). To illustrate the impact of gray area changes on module identification and validation by TBA and SBA, we changed the gray area genes’ expression levels to 0.1, 0.5, 0.9, 1.1, 1.5, and 2 times that of the original datasets to obtain simulated datasets. Based on the simulated datasets, we compared the Variation Ratio (VR, Eq. 3) of TBA (WGCNA) and SBA (pvclust) for module identification and validation.


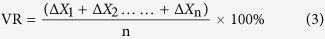


where ΔX is the changed number of modules or genes relative to the original data, and n is the number of the simulated datasets.

## Additional Information

**How to cite this article**: Li, B. *et al.* Quantitative assessment of gene expression network module-validation methods. *Sci. Rep.* **5**, 15258; doi: 10.1038/srep15258 (2015).

## Supplementary Material

supplementary table 1

## Figures and Tables

**Table 1:** Topology- and statistics-based methods for module validation.

No.	Type	Index	Equation	Criteria	Application	Test data	Ref.
Topological validation							
1	Integrated index	Z_summary_	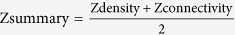	≥10, strongly preserved; 2~10, moderately preserved; ≤2, no preservation	Composite preservation statistics to validate whether a module is significantly preserved in another network. Apply to correlation networks (e.g., co-expression networks)	yes	35,40,41
2		Z_summaryADJ_	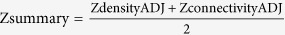	≥10, strongly preserved; 2~10, moderately preserved; ≤2, no preservation	Same as above. Apply to general networks (e.g., adjacency matrix networks)	yes	35
3		medianRank	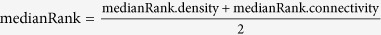	The lower the better	Same as above.	yes	35
4	Single index	Entropy	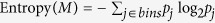	The smaller the better	Access the quality of identified modules. A good quality module is expected to have a low entropy.	no	42,43
5		M_pres_	M_pres_ = cor(k^l^,k^m^)	The closer to 1, the better	Describe the preservation of intra-modular connectivity across two networks. A p-value can be assigned to evaluate the reproducibility of modules.	yes	44,45
6		NB value	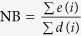	NB ≥ 0.5	A ratio of edges within a module and the total number of edges between modules is used to select modules with high intra-modular connectivity.	no	26
7		CS (S)	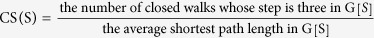	CS (S) > 0, the higher the better	Describe the compactness and neighboring conditions of a cluster. Apply to select good clusters from integrated clustering results	no	46
8		LS (S)	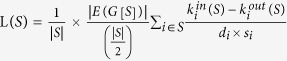	The higher the better	Judge the quality of a cluster S in a graph G and help to select good clusters from integrated clustering results.	no	47
9		Modularity	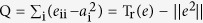	0.3 ≤ Q ≤ 0.7	Evaluate the level of modular structure and the best split of a network into modules.	no	2,39,102
Statistical validation							
1	Integer linear programming	C · (X_1_, X_2_, …, X_k_)	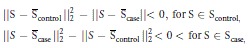	C ≤ 0, the smaller the better	A classifier and integer linear programming model to select modules based on the activity of the module in case and control samples.	yes	49,50
2	Bootstrap resampling	P-value	NULL	P ≤ 0.05	P-value is derived from multiscale bootstrap resampling to assess the uncertainty of clustering analysis and search for significant modules.	no	25,33
3		Consensus score	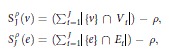	≥ρ, the higher the better	A jackknife resampling procedure is used to assess the accuracy and robustness of functional modules resulting in an ensemble of optimal modules.	no	56
4	Permutation test	Combinatorial p-value	NULL	Combinatorial criteria: (1) P_(Zm)_ < 0.05; (2) P_GL_, P_nSNPs_, P_topo_ < 0.05; (3) P_emp_ < 0.05 Additional criteria: P_(Zm(eval))_ and/or P_emp(eval)_ < 0.05	Significance and permutation tests are used to calculate the P value of module scores. Appropriate for GWAS data; multiple GWAS datasets are needed when using additional criteria.	yes	52
5		coClustering (q)	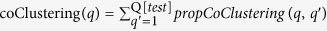	≥95%	A cross-tabulation-based statistic for determining whether modules in the reference dataset are preserved in a test dataset, a permutation test to determine the p value.	yes	45
6	Modular compatibility	Compatibility Score (*Cp)*	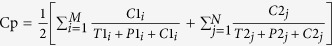	The closer to 1, the better	An indication of agreement or overlap between two sets of modules to measure the network modular compatibility between two networks.	yes	53
7		Matching p-value	NULL	P < 0.05	Modified hypergeometric test-derived p-values with Bonferroni correction to measure modules’ conservation between any two species or networks.	yes	54
8		IGP	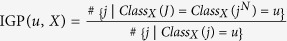	The closer to 1, the better	Defined to validate an individual cluster’s reproducibility and prediction accuracy.	yes	55

The topology-based methods (TBA) and statistics-based methods (SBA) for module validation. The columns reports the types, index names, equations, criteria (the cut-off value to evaluate modules), applicable conditions, test data (whether this method requires an additional test network to validate a module) and references.
