# Supplementary material for: Quantitative assessment of gene expression network module-validation methods
Source: Sci Rep. 2015 Oct 16;5:15258. doi: 10.1038/srep15258 (PMC4607977; doi:10.1038/srep15258)
Supplement: supplementary table 1 [file srep15258-s1.doc]

**Quantitative assessment of genome-wide module-validation methods**

Bing Li,^1,2^ Yingying Zhang,^1^ Yanan Yu,^1^  Pengqian Wang,^1^ Yongcheng Wang,^3^ Zhong Wang,^1,*^ Yongyan Wang^1,*^

^1^ Institute of Basic Research in Clinical Medicine, China Academy of Chinese Medical Sciences, 16 Nanxiaojie, Dongzhimennei, Beijing 100700, China

^2^ Institute of Information on Traditional Chinese Medicine, China Academy of Chinese Medical Sciences, Beijing 100700, China

^3^ Brightech International, LLC, 285 Davidson Ave #504, Somerset, NJ 08873, USA

*Corresponding author: [zhonwtcm@sina.com](mailto:zhonwtcm@sina.com) (Zhong Wang), [wangyongyan@sina.cn](mailto:wangyongyan@sina.cn) (Yongyan Wang).

**Supplementary Table 1 Functional module validation methods**

| **No.** | **Type** | **Index** | **Equation** | **Criteria** | **Application** | **Test data** | **Ref** |
| --- | --- | --- | --- | --- | --- | --- | --- |
| 1 | Functional enrichment  score | ex.mean  cor.mean  mf.mean  bp.mean  cc.mean | NULL | High Confidence: mf.mean,  bp.mean and cc.mean ≧ 0.5; Medium Confidence: bp.mean,cc.mean≥0.5 and cor.mean≥05; Low Confidence: bp.mean,cc.mean≥0.5 and cor.mean≤0.5. | A confidence scoring scheme based on gene expression and GO annotation. | no | 21 |
| 2 |  | L(C) | $\text{L}(C)=Max(\sum_{p_{i}\in T} w_{i}F(p_{i}))$ | L(C)≥0.5 | A likelihood model to evaluate the statistical significance of the disease-related clusters in multiple biological evidences. Apply to select the phenotype-related clusters. | no | 72 |
| 3 | Functional homogeneity | Strength | $\text{Strength}(M)=\max_{i}s_{i}(M)$ | The higher the better | The maximum significance of the associations of the given module over all loci, to qualify the modules association to genomic alterations | no | 42 |
| 4 |  | Specificity | $\text{Specificity}(M)=\sum_{j\in bins}c_{j}/\vert bins\vert$ | The higher the better | Quantifies the distinction between significant associations and the remainder of the loci. | no | 42 |
| 5 |  | p−value | $\text{p-value}=\sum_{\mathrm{i}=\mathrm{x}}^{\mathrm{n}} \frac{(_{\mathrm{i}}^{\mathrm{M}})(_{\mathrm{n}-\mathrm{i}}^{\mathrm{N}-\mathrm{M}})}{_{\mathrm{n}}^{\mathrm{N}}}$ | The smaller the better | The probability of a given module enriched by a given functional group to assess the biological significance of each predicted module. | no | 7,46,70 |
| 6 |  | R score | $\text{R}=1-\left(\frac{-\left(\sum_{s=1}^{n} p_{s}\log_{2}p_{s}\right)}{\log_{2}n}\right)$ | The closer to 1, the better | A scoring system to assess the functional homogeneity of proteins within a predicted cluster. | no | 7,71 |
| 7 | Protein complex matching | F-measure | $\text{F-measure}=\frac{2\times S_{n}\times S_{p}}{S_{n}\times S_{p}}$ | The closer to 1, the better | Estimate how a module is matched to a known protein complex. To assess the biological significance of each module based on protein complex. | no | 44,70,69 |
| 8 |  | OS (PC, KC) | $OS(PC,KC)=\frac{\vert PC\cap KC\vert^{2}}{\vert PC\vert\times\vert KC\vert}$ | OS (PC, KC) > 0.2 | Apply to determine how a predicted module could match a known protein complex from the benchmark set of complexes. | no | 7,44,70,73 |
| 9 | Experimental validation | Genetic interaction | NULL | Module genes have co-transcription or other genetic interactions | RT-qPCR, western blotting, and siRNA knock-down methods to verify the biological interactions. | no | 25,26,28 |
| 10 |  | Coexpression relationship | NULL | Module genes have co-expression relationship | Experiment to verify the co-expression relationship between members of a module. | no | 27,30 |
| 11 |  | Flux coupling  analysis | NULL | Reactions with correlated fluxes | Multivariate statistical analysis of the NMR-derived intra- and extracellular metabolite profiles to verify metabolic network modules. | no | 29 |

**Supplementary Table 1**. The function-based methods for module validation. This column reports the types, index names, equations, criteria (the cut-off value to evaluate modules), applicable conditions, test data (whether this method requires an additional test network to validate a module) and references.

**Supplementary Table 2 The gene expression information and network topological parameters of 10 datasets**

|  | **GSE4882** | **GSE2283** | **GSE6448** | **GSE6738** | **GSE12148** | **GSE12520** | **GSE5373** | **GSE5316** | **GSE29230** | **GSE5834** |
| --- | --- | --- | --- | --- | --- | --- | --- | --- | --- | --- |
| **Organisms** | Homo sapiens | Homo sapiens | Homo sapiens | Homo sapiens | Candidatus Blochmannia floridanus | Homo sapiens | Homo sapiens | Homo sapiens | Mus musculus | Mus musculus |
| **Platform** | GPL3730 | GPL1874 | GPL3632 | GPL4665 | GPL7048 | GPL7199 | GPL4010 | GPL3789 | GPL13520 | GPL4317 |
| **Gene number** | 672 | 448 | 2445 | 872 | 564 | 3040 | 2043 | 2343 | 972 | 3520 |
| **Ref samples** | 60 | 25 | 93 | 14 | 16 | 55 | 296 | 18 | 42 | 20 |
| **Test samples** | 60 | 25 | 92 | 14 | 16 | 54 | 296 | 17 | 41 | 19 |
| **Clustering coefficient** | 0.484 | 0.624 | 0.643 | 0.703 | 0.177 | 0.583 | 0.715 | 0.513 | 0.676 | * |
| **Connected components** | 6 | 3 | 28 | 2 | 52 | 2 | 2 | 2 | 2 | * |
| **Network diameter** | 10 | 7 | 18 | 6 | 19 | 4 | 4 | 3 | 6 | * |
| **Network centralization** | 0.258 | 0.244 | 0.031 | 0.36 | 0.04 | 0.171 | 0.485 | 0.38 | 0.163 | * |
| **Shortest paths** | 359410（96%） | 191410（98%） | 4058640（85%） | 756032（99%） | 14654  （20%） | 9135508（99%） | 4122932（99%） | 4633258（99%） | 845482  (99%) | * |
| **Characteristic path length** | 3.058 | 2.37 | 6.694 | 1.927 | 6.861 | 2.246 | 1.892 | 1.931 | 2.639 | * |
| **Avg.number of neighbors** | 26.344 | 61.023 | 17.216 | 226.525 | 2.347 | 210.906 | 393.52 | 231.962 | 65.607 | * |
| **Number of nodes** | 610 | 442 | 2183 | 872 | 271 | 3025 | 2033 | 2155 | 922 | * |
| **Network density** | 0.043 | 0.138 | 0.008 | 0.26 | 0.009 | 0.07 | 0.194 | 0.108 | 0.071 | * |
| **Network heterogeneity** | 1.216 | 0.7 | 0.955 | 0.544 | 0.892 | 0.516 | 0.794 | 0.483 | 0.661 | * |
| **MCL modularity** | 0.2585 | 0.0084 | 0.9144 | 0.0019 | 0.834 | 0.5084 | △ | 0.3332 | 0.6233 | * |
| **QCUT modularity** | 0.4028 | 0.4024 | 0.9254 | 0.3507 | 0.847 | 0.5632 | △ | 0.4478 | 0.6578 | * |
| **HQCUT modularity** | 0.4009 | 0.4017 | 0.8406 | 0.3474 | 0.831 | 0.5051 | △ | 0.4401 | 0.6124 | * |

All network parameters and modularity computing were conducted by Cytoscape plugin on an Intel Xeon CPU with 8 GB of memory.

* Execution times longer than 72 hours with no progress, so the operation was aborted.

△ error reported in the process of operation and interrupted.

**Supplementary Table 2**. The fundamental information and network topological parameters of 10 datasets downloaded from the GEO database (http://www.ncbi.nlm.nih.gov/geo/). Each row shows the basic information of these datasets, including organisms, platforms, the total gene numbers, reference samples (the number of samples used for the reference set), test samples (the number of samples used for test set), clustering coefficient, connected components, network diameter, network centralization, shortest paths, characteristic path length, the average number of neighbors, number of nodes, network density, network heterogeneity, and modularity (the MCL, QCUT and HQCUT methods were used in the CommFinder plugin in Cytoscape).
